# Supplementary material for: Modeling glioblastoma heterogeneity as a dynamic network of cell states
Source: Mol Syst Biol. 2021 Sep 16;17(9):e10105. doi: 10.15252/msb.202010105 (PMC8444284; doi:10.15252/msb.202010105)
Supplement: Supplementary file 6 — Source Data for Figure 5 [file MSB-17-e10105-s004.zip › Figure5A_sourcedata/GSEA_3017/hallmarks_stateF.GseaPreranked.1621934628066/HALLMARK_KRAS_SIGNALING_UP.html]

Details for gene set HALLMARK\_KRAS\_SIGNALING\_UP[GSEA]

|  || Dataset | state33017 |
| Phenotype | NoPhenotypeAvailable |
| Upregulated in class | na\_pos |
| GeneSet | HALLMARK\_KRAS\_SIGNALING\_UP |
| Enrichment Score (ES) | 0.44275847 |
| Normalized Enrichment Score (NES) | 1.9411131 |
| Nominal p-value | 0.0018315018 |
| FDR q-value | 0.00838758 |
| FWER p-Value | 0.083 |
Table: GSEA Results Summary

  

Fig 1: Enrichment plot: HALLMARK\_KRAS\_SIGNALING\_UP      
 Profile of the Running ES Score & Positions of GeneSet Members on the Rank Ordered List

  

| PROBE | GENE SYMBOL | GENE\_TITLE | RANK IN GENE LIST | RANK METRIC SCORE | RUNNING ES | CORE ENRICHMENT || 1 | SPARCL1 |  |  | 6 | 1.804 | 0.1295 | Yes |
| 2 | CPE |  |  | 29 | 1.046 | 0.1963 | Yes |
| 3 | SPP1 |  |  | 57 | 0.898 | 0.2499 | Yes |
| 4 | SCG3 |  |  | 130 | 0.698 | 0.2687 | Yes |
| 5 | TSPAN7 |  |  | 148 | 0.671 | 0.3102 | Yes |
| 6 | PEG3 |  |  | 162 | 0.645 | 0.3516 | Yes |
| 7 | PSMB8 |  |  | 230 | 0.568 | 0.3632 | Yes |
| 8 | CA2 |  |  | 243 | 0.558 | 0.3987 | Yes |
| 9 | ID2 |  |  | 366 | 0.483 | 0.3792 | Yes |
| 10 | CROT |  |  | 460 | 0.447 | 0.3703 | Yes |
| 11 | TFPI |  |  | 531 | 0.421 | 0.3696 | Yes |
| 12 | HDAC9 |  |  | 579 | 0.404 | 0.3782 | Yes |
| 13 | APOD |  |  | 636 | 0.389 | 0.3815 | Yes |
| 14 | GPRC5B |  |  | 653 | 0.385 | 0.4025 | Yes |
| 15 | RGS16 |  |  | 691 | 0.373 | 0.4132 | Yes |
| 16 | WNT7A |  |  | 735 | 0.362 | 0.4205 | Yes |
| 17 | MAP7 |  |  | 745 | 0.359 | 0.4428 | Yes |
| 18 | EVI5 |  |  | 994 | 0.310 | 0.3541 | No |
| 19 | CXCR4 |  |  | 1003 | 0.309 | 0.3732 | No |
| 20 | ADAM17 |  |  | 1215 | 0.277 | 0.2988 | No |
| 21 | SDCCAG8 |  |  | 1291 | 0.269 | 0.2848 | No |
| 22 | PRRX1 |  |  | 1341 | 0.262 | 0.2820 | No |
| 23 | ITGA2 |  |  | 1395 | 0.257 | 0.2771 | No |
| 24 | HBEGF |  |  | 1583 | -0.282 | 0.2137 | No |
| 25 | EMP1 |  |  | 1598 | -0.285 | 0.2284 | No |
| 26 | TMEM158 |  |  | 1803 | -0.362 | 0.1633 | No |
| 27 | NIN |  |  | 2032 | -0.523 | 0.0992 | No |
Table: GSEA details [plain text format]

  

Fig 2: HALLMARK\_KRAS\_SIGNALING\_UP: Random ES distribution      
 Gene set null distribution of ES for **HALLMARK\_KRAS\_SIGNALING\_UP**
